# Supplementary material for: Open Source Repository and Online Calculator of Prediction Models for Diagnosis and Prognosis in Oncology
Source: Biomedicines. 2022 Oct 23;10(11):2679. doi: 10.3390/biomedicines10112679 (PMC9687260; doi:10.3390/biomedicines10112679)
Supplement: Supplementary file 1 [file biomedicines-10-02679-s001.zip › biomedicines-1936626-supplementary.pdf]

Figure S1

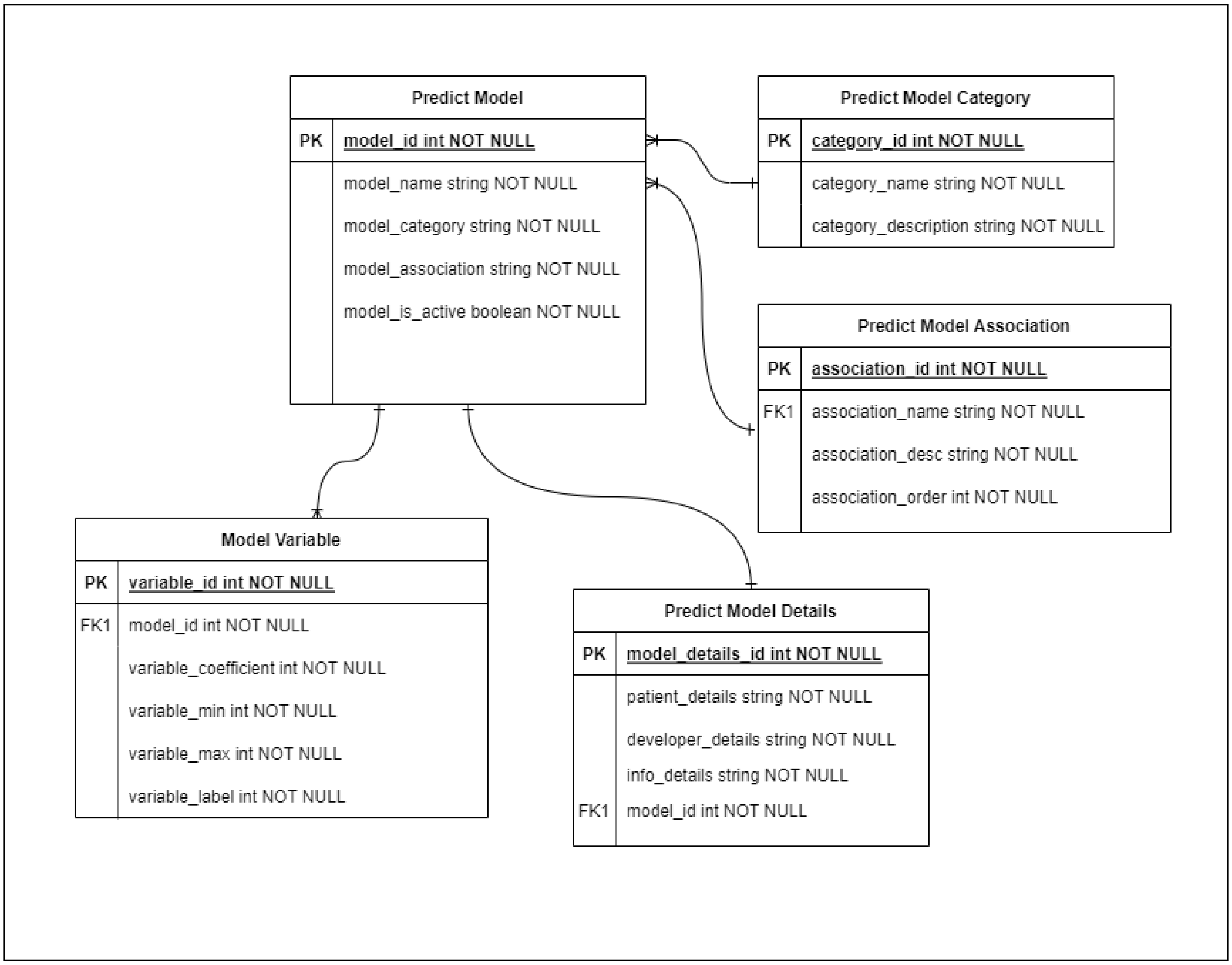

Above displayed is the ER Diagram for the Database of the Ai4Cancer web application. There, the main table where the predict model information is stored is the **Predict Model** table. Each of these models have a category as well as an association. Some examples for the Model Categories are Logistic Regression Model, Linear Regression Model, Segmentation Model, Custom Model etc., which are stored in the **Predict Model Category** table. This category information is then used when determining the type of calculation carried out by the model. Examples for the Model Associations are Brain, Endometrium, Head & Neck, Lung etc. which are used to sort these models in different parts of the body for ease of access when browsing the models. This data is stored in the **Predict Model Association** table

Each predict model is connected to a **Predict Model Detail** table, which records information about the details of the model developer, intended patients, information required to process the model etc. These details are also used when displaying the model details on the web application. Finally, each Model is also connected to the **Model Variable** table, where the variables used to calculate the model are defined. When a particular user submits the model details, these tables will be filled automatically and subsequently be used when displaying and testing out the model.
